# Autologous stem cell transplantation following simultaneous liver and kidney transplantation in severe amyloid light chain amyloidosis associated with multiple myeloma: a case report

**DOI:** 10.1186/s13256-020-02511-9

**Published:** 2020-10-25

**Authors:** R. Al-Zoairy, A. Viveiros, H. Zoller, S. Schneeberger, G. Oberhuber, E. Gunsilius, H. Tilg, D. Wolf, J. D. Rudzki

**Affiliations:** 1 Department of Internal Medicine I (Gastroenterology, Hepatology, Endocrinology and Metabolism), Medical University of Innsbruck, Innsbruck, Austria; 2 Department of Visceral, Transplant and Thoracic Surgery, Medical University of Innsbruck, Innsbruck, Austria; 3 INNPATH, Pathology Service for the Medical University of Innsbruck, Innsbruck, Austria; 4 Department of Internal Medicine V (Hematology & Oncology), Medical University of Innsbruck, Innsbruck, Austria

**Keywords:** AL amyloidosis, Multiple myeloma, Transplantation, Case report

## Abstract

**Introduction:**

The involvement of vital organs in multiple myeloma (MM) with systemic amyloid light-chain (AL) amyloidosis can lead to acute organ failure. In this case, the fear of recurrence or progression of multiple myeloma often excludes those patients from undergoing organ transplantation. Nevertheless, clinically fit patients might benefit from a different therapeutic approach. This case presentation might highlight this particular unmet need and strengthen a different treatment approach.

**Case presentation:**

To our knowledge, we present the first case of successful simultaneous liver and kidney transplantation, followed by autologous stem cell transplantation in a 60-year-old Caucasian male patient suffering from MM (Durie-Salmon stage IIB; ISS-stage: III, RISS stage: III) with primary AL amyloidosis. Chemotherapy treatment led to end-stage kidney disease requiring dialysis. Liver failure also occurred after at least three cycles of CyBorD (bortezomib, cyclophosphamide, and dexamethasone) of induction therapy with a good hematologic response. Over three years after the initial diagnosis, the patient is reportedly showing an excellent quality of life and a complete remission.

**Discussion and Conclusion:**

We conclude that kidney and liver transplantation followed by autologous stem cell transplantation can be a treatment option for a selected group of patients with MM if AL amyloidosis is leading. In the end, the remission assessment by IMWG response criteria displayed a complete remission of MM together with complete reconstitution of organ functions (liver & renal function) as long as upfront clinical evaluation excludes significant cardiac involvement and other severe co-morbidities.

## Background

Systemic amyloid light chain amyloidosis (AL) leading to amyloid deposition and functional damage in various vital organs is a common complication in patients with multiple myeloma (MM). The most frequently affected organs are kidney, heart, and liver [1]. Standard first-line therapy comprises immune modulatory drugs combined with proteasome inhibitors and corticosteroids (dexamethasone). In cases where this elicits a response, this therapy is followed by stem cell mobilization (SCM), myeloablative chemotherapy (high-dose melphalan), and subsequent autologous stem cell transplantation (ASCT) [2]. However, therapy options are often limited by the severity and type of organ damage. In acute organ failure, patients are rarely considered for organ transplantation owing to the fear of progression of myeloma or its recurrence under chronic immunosuppression [3].

Here, we report what is, to the best of our knowledge, the first case of successful simultaneous liver and kidney transplantation followed by ASCT in MM and AL amyloidosis. The patient had International Staging System (ISS) stage III MM with severe AL amyloidosis (revised prognostic staging system for light chain amyloidosis incorporating cardiac biomarkers and serum free light chain measurements [Mayo Score] III; hepatic, renal, and heart involvement), resulting in end-stage liver and kidney disease subsequently requiring dialysis.

## Case presentation

In August 2016, a 60-year-old Caucasian man was referred from a secondary care center to the University Hospital of Innsbruck, Austria, with the following symptoms: fatigue, weight loss of about 5 kg in the previous 6–9 months, unclear hepatopathy, and kidney failure. Physical examination at admission showed hepatomegaly but no other pathological findings.

His medical history included hypothyroidism caused by autoimmune thyroiditis and arterial hypertonia but no previous report of liver or kidney disease. He presented with an excellent Karnofsky performance status of 100% (according to Eastern Cooperative Oncology Group 0).

The laboratory parameters at admission are listed in Table 1. A complete blood count and serum electrolyte results were within the normal ranges. Serological tests for hepatitis B, hepatitis C, and human immunodeficiency virus were negative.

**Table 1:** Laboratory values at admission, at the time of high urgency listing for transplantation and over 3 years after first being diagnosed with amyloid light chain amyloidosis and multiple myeloma

Parameter	At admission	At time of high urgency listing	3 years after diagnosis	Reference values
**Creatinine (mg/dl)**	**2.09**	**3.07**	**1.62**	0.67–1.17
**Total bilirubin (mg/dl)**	0.76	**33.18**	0.96	0.00–1.28
**AST (U/l)**	**72**	**88**	32	10–50
**ALT (U/l)**	**54**	43	31	10–50
**γ-GT (U/l)**	**1270**	**89**	**161**	10–71
**AP (U/l)**	**676**	**136**	**332**	40–130
**INR**	1	**1.7**	0.9	0.85–1.27
**hsTropT (ng/l)**	**31.4**	n/a	**23.5**	0.0–14
**NT-proBNP (ng//l)**	**1428**	n/a	**306**	39–190
**Lambda LC (mg/l)**	**262**	n/a	7.1	5.7–26.3
**Kappa LC (mg/l)**	**40.7**	n/a	11.2	3.3-19.4
**FLC ratio**	**0.16**	n/a	1.58	0,26-1,65
**M gradient**	2.05	n/a	Not detectable –immunofixation negative	
**β2 microglobulin (mg/l)**	**8.1**	n/a	**4.4**	1.1-2.5
**RBC (g/dl)**	13.4	n/a	13.7	13.0-17.7

Pathological values are in bold

*γ-GT* γ-glutamyltransferase, *ALT* Alanine amino-transferase, *AP* Alkaline phosphatase, *AST* Aspartate amino-transferase, *FLC* free light chain, *hsTropT* highly sensitive Troponin T, *INR* international normalized ratio, *LC* light chain, *M gradient* Serum-electrophoresis absolute amount, *n/a* not applicable, *NT-proBNP* N-terminal-pro B-type natriuretic peptide, *RBC* Red blood cells

Quantitative immunoglobulin (Ig) assay of the blood revealed an elevation of IgG with 2600 mg/dL (670–1840 mg/dL) and reduced values of IgA with 55 mg/dL (103–595 mg/dL) and IgM with 20 mg/dL (36–238 mg/dL). Serum immunofixation confirmed IgG gammopathy with lambda light chain and Bence–Jones lambda expression. Immunofixation of the urine revealed excretion of lambda light chain and Bence–Jones lambda.

Abdominal sonography showed hepatomegaly, minor amounts of ascites, and signs of diffuse renal parenchymal damage. Because cardiac involvement was suspected owing to the slightly elevated high-sensitivity troponin T, cardiac magnetic resonance imaging (MRI) and echocardiography were performed. The MRI suggested cardiac involvement, whereas the echocardiography repeatedly showed normal cardiac function. Therefore, no myocardial biopsy was carried out. The first bone computed tomography (CT) scan revealed scattered bilateral osteolytic lesions along the ribs.

A bone marrow biopsy revealed an infiltration of clonal IgG lambda-expressing plasma cells with a cellularity of 20%. Cytometry analysis showed the following: 7% clonal plasma cells (all tested markers are listed in detail in Table 2). Cytogenetic testing demonstrated a deletion in 13q in 98% and translocation in (4;14) in 89.8% (Table 2).

**Table 2:** Results of bone marrow biopsy, fluorescence-activated cell sorting and cytogenetics

Test	Finding
**Histology**	20% clonal plasma cells lambda
**FACS**	7% clonal plasma cells, CD38+, CD138+, CD56+, CD27+, CD200+, cytkappa+, cytlambda+, CD19−, CD45−, CD117−, CD28−, CD81−
**Cytogenetics**	del13q 98%, t(4;14) 89.8%

**Fig. 1:**
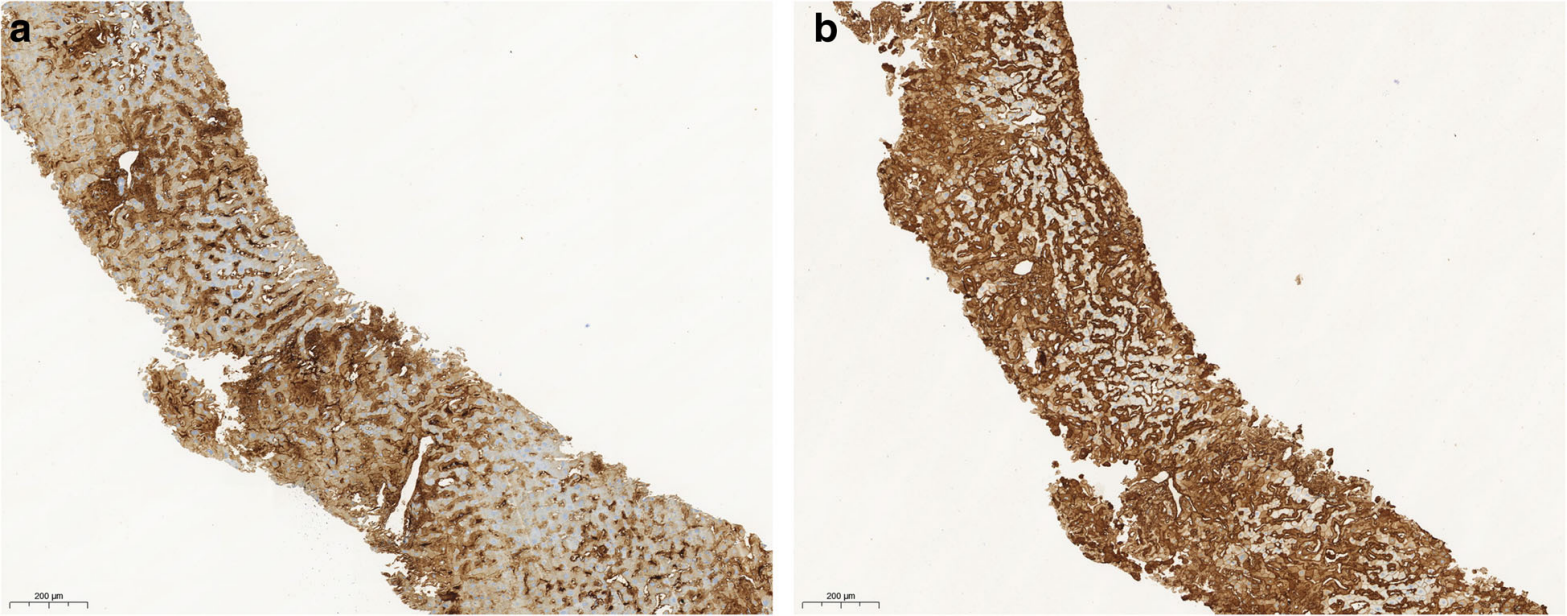
Liver biopsy and immunohistochemistry. **a** Amyloid P and **b** lambda light chain. Both are colored brown and typically localized near to each other (compare the two pictures)

Hepatic venous pressure gradient measurement with a trans-jugular liver biopsy and gastroscopy revealed a clinically significant portal hypertension (pressure gradient of 30 mmHg) with small esophageal varices. The A liver biopsy confirmed extensive deposition of AL amyloid of lambda light chain type (Fig. 1).

A kidney biopsy was not performed.

Consequently, the patient was diagnosed with MM Durie–Salmon stage IIB (ISS III, R-Revised ISS [R-ISS] III) with the leading disease of systemic AL amyloidosis (Mayo Sscore III). The systemic AL amyloidosis manifested itself as an advanced disease with end-stage liver and kidney disease as well as a suspected heart involvement (as per the MRI) without functional impairment.

Therefore, treatment with bortezomib, cyclophosphamide, and dexamethasone (CyBorD) was started, following a standard protocol for systemic AL amyloidosis [4]. At the same time, kidney function deteriorated further, which resulted in end-stage kidney disease requiring dialysis.

The first two chemotherapy treatment cycles resulted in a very good partial remission (VGPR). During the third cycle, the patient developed jaundice and his liver function rapidly worsened, culminating in acute liver failure in November 2016. Immediate intensive care treatment rescued the patient, but owing to the clinical situation the hematologic treatment could not be continued.

After development of progressive hepatic encephalopathy, acute liver failure was stated. As a consequence, an interdisciplinary team of clinicians decided to list the patient for high urgency liver and kidney transplantation with a Model for End-Stage Liver Disease score of 39 (exact laboratory values are listed in Table 1).

Ten days after enrolment on the high-urgency waiting list, simultaneous liver and kidney transplantation was performed in December 2016 and triple immunosuppression was started with tacrolimus, mycophenolate, and prednisolone.

**Fig. 2:**
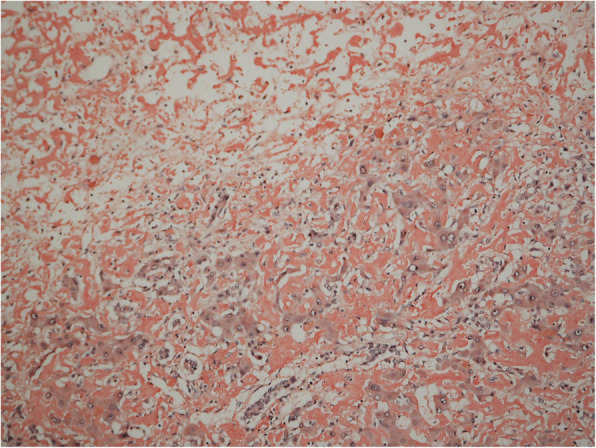
Explanted liver with Congo Red stain, × 400 magnification. Congo Red highlights an area with a diffuse sinusoidal pattern of hepatic amyloidosis. In the upper third hepatocytes are necrotic

The histology of the explanted liver confirmed hepatic amyloidosis but also revealed signs of major toxic damage, likely caused by chemotherapy. Although a sinusoidal pattern is more common in AL amyloidosis and a vascular pattern in amyloid A amyloidosis, distribution patterns of amyloid deposition revealed an overlap of both (Fig. 2).

Two months after solid-dual organ transplantation (kidney and liver), hematopoietic SCM and apheresis were performed using a standard protocol of SCM and subsequent ASCT after reduced-dose Melphalan conditioning chemotherapy (140 mg/m^2^) [5]. During the ASCT treatment process, immunosuppression with tacrolimus and prednisolone was reduced, whereas the mycophenolate dose remained unchanged.

The following course proceeded without any further complications and a complete hematologic remission was achieved (M-gradient beneath limit of detection and β-2-microglobuline consistently falling; Table 1).

Without any rational explanation, a bone CT scan performed in November 2017 could not confirm the initially found osteolytic lesions. Owing to increasing cholestasis parameters (bilirubin, alkaline phosphatase, γ-glutamyltransferase) in December 2017, endoscopic retrograde cholangiography was performed. An anastomosis stenosis (AS) and a non-anastomosis stenosis (NAS) were found and were repeatedly treated with balloon dilatation and plastic stents.

At the most recent follow-up, over 3 years after simultaneously diagnosed extended AL amyloidosis and the first diagnosis of MM, the patient demonstrated the following response: a complete hematologic remission without a detectable M-gradient in serum electrophoresis and negative immune fixation, good liver function, and slightly impaired renal function under immunosuppression with tacrolimus (exact parameters are listed in Table 1). Finally, an AS and a NAS were treated with balloon dilatation and placement of two plastic stents, which are now permanently removed. The patient reported having an excellent quality of life. He returned to work in his profession and is able to participate in hiking trips with his family.

## Discussion and conclusion

AL amyloidosis is a systemic disorder characterized by the deposition of amyloid fibrils that can affect any organ, such as skin, gastrointestinal tract, heart, lungs, liver, and kidney [1]. The kidney is one of the most frequently affected organs in amyloidosis. Amyloid deposits often disturb physiological organ structures and cause end-stage renal disease, requiring dialysis [6].

Hepatic involvement is also common but in the majority of cases characterized by only mild clinical alterations such as manifestation of hepatomegaly and/or slightly elevated liver function test results. However, the appearance of jaundice, as a sign of cholestatic amyloidosis, results in a very poor prognosis [7, 8].

Cardiac involvement is also well described; if it occurs, involvement of this particular organ is usually the main determinant of patient survival [9, 10].

In the past, patients with AL amyloidosis and MM usually had no access to solid organ transplantations because of poor overall survival. However, owing to new therapeutic options, including the introduction of second-generation proteasome inhibitors [11–14], second- or third-generation immunomodulatory imide drugs (IMid) [15, 16], and anti-CD38 antibodies like daratumumab [17, 18], the prognosis of these patients has significantly improved in both entities. Prior treatment of newly diagnosed MM with these new substances results in better progression-free survival and overall survival rates. Additionally, even patients in relapse/refractory settings have vastly improved outcomes that have translated into mighty advances in disease control and, subsequently, survival.

Sattianayagam *et al.* [19] reported on 22 renal, 14 heart, and 9 liver transplantations performed between 1984 and 2009 in patients with AL amyloidosis who were registered at the UK National Amyloidosis Centre. The group found a 5-year overall survival of 67% for renal, 45% for heart, and 22% for liver transplant recipients. No transplant failed due to amyloid recurrence, despite evidence of amyloid within the allografts. However, patients treated in the pre-bortezomib era were also included [19], suggesting that survival might even be superior with the currently available treatment regimens.

Lum *et al.* [20] reported two cases of successful kidney transplantation without subsequent ASCT and only partial MM remission under ongoing bortezomib therapy. Therefore, according to Bridoux *et al.* [6], it is reasonable to perform renal transplantation in patients with AL amyloidosis and a good response to bortezomib-based therapies, in particular when subsequent ASCT is performed.

In our presented case, suppression of the underlying light chain-producing plasma cell clone was achieved with a short induction therapy regimen of three incomplete cycles of CyBorD, subsequent reduced dose melphalan conditioning treatment (70% dosage), and ASCT [6].

There are only a few cases reports on patients with AL amyloidosis who underwent liver transplantation. The first case was reported by Sandberg-Gertzen *et al.* [21] in the pre-bortezomib era, but with recurrent amyloidosis in the allograft within 1 year. Elnegouly *et al*. [22] reported a successful liver transplantation and subsequent ASCT in a patient with a similar setting of MM and AL amyloidosis who was treated with bortezomib [22]. Ueno *et al*. [3] showed a good outcome in liver amyloidosis even without ASCT with no disease progression or graft-related problems within the observation period of 26 months. Furthermore, double ASCT and liver transplantation during a follow-up period of 8 years was associated with beneficial outcomes [23]. Dispenzieri *et al*. [24] described superior outcomes in patients with AL amyloidosis undergoing ASCT compared with other patients with MM.

Our patient was admitted with end-stage renal disease leading to renal replacement therapy and liver amyloidosis with elevated liver enzymes but without signs of acute liver failure. Acute liver failure only occurred after two complete and one incomplete third cycle of CyBorD, despite a good hematologic response to therapy (VGPR). Therefore, an additional hepatotoxic effect of CyBorD as a cause of the rapid deterioration of liver function was discussed prior to the listing for simultaneous liver and kidney transplantation. The histology of the explanted liver supported that notion (Fig. 2).

Bortezomib can cause mild liver injury with slightly elevated liver enzymes, whereas cases with acute hepatic necrosis and cholestatic hepatitis are rare but have been reported previously [25]. Mild elevation of liver enzymes is also common for cyclophosphamide and in rare cases can lead to acute liver failure [26].

In summary, our case shows that kidney and liver transplantation followed by ASCT can be a treatment option in a selected group of patients with MM in the event of simultaneously diagnosed AL amyloidosis of a very extended degree. However, good hematologic response to induction chemotherapy, no significant functional cardiac involvement, and the exclusion of severe comorbidities should be ensured.

To our knowledge this is the first reported case of a successful simultaneous liver and kidney transplantation followed by reduced high-dose melphalan (70% dosage) conditioning therapy plus subsequent ASCT in a patient with systemic AL amyloidosis of a very extended degree accompanied by the diagnosis MM of IgG lambda subtype resulting in complete remission for more than 3 years without the need for an IMid-based maintenance therapy.

## Data Availability

Not applicable.

## Abbreviations

AL: Amyloid light chain
AS: Anastomosis stenosis
ASCT: Autologous stem cell transplantation
CyBorD: Bortezomib, cyclophosphamide and dexamethasone
FLC: Free light chains
FACS: Fluorescence-activated cell sorting
hsTropT: Highly sensitive Troponin T
Ig: Immunoglobulin
IMid: Immunomodulatory imide drugs
ISS: International Staging System
Mayo Score: Revised prognostic staging system for light chain amyloidosis incorporating cardiac biomarkers and serum free light chain measurements
MM: Multiple myeloma
NAS: Non-anastomosis stenosis
NT-proBNP: N-terminal- pro-B-type natriuretic peptide
R-ISS: Revised International Staging System
SCM: Stem cell mobilization
VGPR: Very good partial response
